# An Easy and Efficient Strategy for the Enhancement of Epothilone Production Mediated by TALE-TF and CRISPR/dcas9 Systems in *Sorangium cellulosum*

**DOI:** 10.3389/fbioe.2019.00334

**Published:** 2019-11-26

**Authors:** Wei Ye, Taomei Liu, Muzi Zhu, Weimin Zhang, Zilei Huang, Saini Li, Haohua Li, Yali Kong, Yuchan Chen

**Affiliations:** State Key Laboratory of Applied Microbiology Southern China, Guangdong Provincial Key Laboratory of Microbial Culture Collection and Application, Guangdong Open Laboratory of Applied Microbiology, Guangdong Institute of Microbiology, Guangdong Academy of Sciences, Guangzhou, China

**Keywords:** *Sorangium cellulosum*, TALE-TF, CRISPR/dCas9 activation, epothilones, regulatory mechanism

## Abstract

Epothilones are a kind of macrolides with strong cytotoxicity toward cancer cells and relatively lower side effects compared with taxol. Epothilone B derivate ixabepilone has been used for the clinical treatment of advanced breast cancer. However, the low yield of epothilones and the difficulty in the genetic manipulation of *Sorangium cellulosum* limited their wider application. Transcription activator-like effectors-Trancriptional factor (TALE-TF)-VP64 and clustered regularly interspaced short palindromic repeats (CRISPR)/dCas9-VP64 have been demonstrated as effective systems for the transcriptional improvement. In this study, a promoter for the epothilone biosynthesis cluster was obtained and the function has been verified. The TALE-TF-VP64 and CRISPR/dcas9-VP64 target P3 promoter were electroporated into *S. cellulosum* strain So ce M4, and the transcriptional levels of epothilone biosynthesis-related genes were significantly upregulated. The yield of epothilone B was improved by 2.89- and 1.53-fold by the introduction of recombinant TALE-TF-VP64-P3 and dCas9-VP64-P3 elements into So ce M4, respectively. The epothilone D yield was also improved by 1.12- and 2.18-fold in recombinant dCas9-So ce M4 and TALE-VP64 strains, respectively. The transcriptional regulation mechanism of TALE-TF-VP64 and the competition mechanism with endogenous transcriptional factor were investigated by electrophoretic mobility shift assay (EMSA) and chromatin immunoprecipitation (ChIP), demonstrating the combination of the P3 promoter and TALE-TF element and the competition between TALE-TF and endogenous transcriptional protein. This is the first report on the transcriptional regulation of the epothilone biosynthetic gene cluster in *S. cellulosum* using the TALE-TF and dCas9-VP64 systems, and the regulatory mechanism of the TALE-TF system for epothilone biosynthesis in *S. cellulosum* was also firstly revealed, thus shedding light on the metabolic engineering of *S. cellulosum* to improve epothilone yields substantially and promoting the application of epothilones in the biomedical industry.

## Introduction

Epothilones are 16-member macrolides with strong anticancer activity and produced by *Sorangium cellulosum*. Epothilones show stronger anticancer effects, broader spectrum of antitumor activity, simpler structure, lower side effects, and much better water solubility than the popular anticancer drug taxol (Bollag et al., 1995; Gerth et al., 1996; Höfle et al., 1996). Taxol yield has fallen sharply because *Taxus* has become increasingly rare, and the high cost of producing taxol through total synthesis has expanded the demand for alternative drugs. Epothilone B derivate ixabepilone has been approved in the clinical treatment of advanced breast cancer, and epothilone B and epothilone D and their derivatives show great potential in cancer treatment, especially for patients with taxane-resistant metastatic breast cancer (Roché et al., 2007; Thomas et al., 2007; Sparano et al., 2010). Epothilones have been considered an ideal substitute for taxol. However, epothilone yield remains low because of the difficulty in genetically manipulating *S. cellulosum* and the cytotoxicity of epothilones during the heterologous expression of epothilone biosynthesis cluster. The low yield of epothilones has limited their application in cancer treatment, and methods for enhancing epothilone yield and optimizing cultivation conditions are limited and random (Ye et al., 2016). Epothilone biosynthesis genes have been successively expressed in some bacteria, including *Myxococcus xanthus* and *Burkholderiales* sp. However, the improvement of epothilone yield through the heterologous expression of epothilone biosynthetic gene clusters is limited because of the toxicity of epothilone toward heterologous hosts. Epothilone yield in native host *S. cellulosum* is usually higher than heterologous hosts because of the high tolerance of *S. cellulosum* to the cytotoxicity of epothilones. Therefore, *S. cellulosum* is the best candidate for the expression of epothilone biosynthetic gene cluster. The scarcity of a genetic manipulation system for *S. cellulosum* impedes progress in the genetic engineering of epothilone biosynthesis in *S. cellulosum*. The transcription enhancement of epothilone biosynthetic cluster is an effective approach for improving epothilone yield. However, there are still few reports on the epothilone yield improvement in *S. cellulosum* by transcriptional activation approaches. Hence, improving epothilone yield by genetically engineering *S. cellulosum* through the transcriptional enhancement of epothilone biosynthetic cluster is urgent.

The epothilone biosynthesis cluster of *S. cellulosum* is a 56-kb gene cluster (Julien et al., 2000; Molnár et al., 2000). Different approaches have been adopted to enhance epothilone yield. Gong et al. (2007) fused the proplasts of different epothilone-producing strains to enhance epothilone B yield to 45.2 mg/L. Meng et al. (2010) improved epothilone B yield in *S. cellulosum* strain ATCC15384 from 5 to 9 mg/L by optimizing fermentation conditions. The biosynthetic cluster of epothilones was identified and heterologously expressed in *Streptomyces coelicolor*, and epothilones A and B were produced with very low yield (Tang et al., 2000). The whole gene cluster of *S. cellulosum* So0157-2 including the promoter sequence was inserted into *M. xanthus* by transposition insertion (Han et al., 2013; Zhu et al., 2015). Epothilone biosynthetic cluster was expressed in *M. xanthus*, and the *epoK* gene, which is responsible for the transformation of epothilone D to epothilone B, was disrupted for the increase of epothilone D yield to 20 mg/L (Julien and Shah, 2002). The CRISPR/Cas9 system is an effective tool for the specific regulation of gene expression levels (Alessandro et al., 2014). CRISPR/dCas9 technology was employed in *M. xanthus* to improve the transcription of heterologously expressed biosynthetic genes for the production of epothilones, and the yield of epothilone B was significantly improved (Peng et al., 2018). The promoters and transcriptional regulatory proteins of epothilone biosynthesis cluster have been investigated. The different promoters of epothilone biosynthetic clusters from *S. cellulosum* were identified and found to have high similarity but very different activities; thus, these promoters are important targets for the regulation of epothilone biosynthesis (Zhu et al., 2013). Proteins binding to the promoters of epothilone biosynthetic clusters were isolated from *S. cellulosum* strain So 0157-2, and their functions were verified by electrophoretic mobility shift assay (EMSA). Transcription activator-like effectors (TALEs) are DNA-binding proteins with high specificity and derived from *Xanthomonas* sp. (Joung and Sander, 2013). A TALE could be combined with transcriptional activation factor (TALE-TF) including VP64 to enhance the transcription level of target genes. The TALE-TF (Perez-Pinera et al., 2013; Hu et al., 2014; Uhde-stone et al., 2014) and CRISPR/dCas9 system (Gao et al., 2013; Qi et al., 2013; Chavez et al., 2015) have been widely used in the enhancement of target genes in mammalian cells. In this study, the promoter P3 of the epothilone biosynthetic cluster in *S. cellulosum* strain So ce M4 was cloned, and the function was validated by detecting luciferase activity. Recombinant TALE-TF and CRISPR/dCas9 elements targeting the P3 promoter were introduced into *S. cellulosum* strain So ce M4 for the upregulation of the transcriptional levels of epothilone biosynthetic genes and enhancement of epothilone yields. This study was the first report on the transcriptional regulation of epothilone biosynthetic cluster in *S. cellulosum* strain through the TALE-TF technology and CRISPR/dCas9 systems, and this is also the first investigation on the transcriptional mechanism of epothilone biosynthetic gene cluster that employs TALE-TF elements by using EMSA and chromatin immunoprecipitation (ChIP) in *S. cellulosum*; hence, this study is of significance for the epothilone yield improvement in *S. cellulosum* So ce M4 by transcriptional regulation approaches. Our investigation provided the molecular clues for the future transcriptional regulation of epothilone biosynthesis in *S. cellulosum* to improve the biosynthesis efficiency of epothilones in *S. cellulosum*, thereby expanding the application of epothilones in cancer treatment.

## Materials and Methods

### Materials

*S. cellulosum* strain So ce M4 (accession no. GQ845309) was isolated from the soil of Xinyi City, Guangdong Province. A FastTALE^TM^ assembly kit including the Ptalen R36 vector was purchased from Sidansai (Shanghai, China). PLX-sgRNA and dCas9-VP64 were purchased from addgene (USA). *S. cellulosum* was cultured in a G52 medium, and the growth temperature was 28°C.

### Amplification and Identification of the P3 Promoter

The P3 promoter was amplified with primers P3 F: TGGCGTCGGGCGCGGGGTCG and P3 R: CCACTCGACCCCGCGCCCGAC to amplify the promoter for epothilone biosynthesis in So ce M4, and the genomic DNAs of So ce M4, So ce M1, and So ce M6 were used as templates. The P3 promoter was then ligated into the pMD18-T vector, and the positive clone was selected with an LB plate containing 100 μg/ml ampicillin and then sequenced. The P3 promoter and pgpd promoter were inserted into the pGL3-Basic vector by using restriction sites *Xho*I and *Hin*dIII to initiate the luciferase expression. The recombinant vectors pGL3-P3, pGL3-pgpd, and pGL3-Basic were transformed into the BL21 (DE3) strain. The bacteria were collected and sonicated, and the supernatants after centrifugation under 8,000 rpm for 10 min were collected and then incubated with luciferin agent for 5 min in equal volume following the instructions of Bright-Lumi^TM^. Firefly Luciferase Reporter Gene Assay Kit (Beyotime, Shanghai, China), and the fluorescence intensities of different supernatants with luciferin agent were detected by an RF-5301PC fluorophotometer (SHIMADZU, Japan).

### Construction of Plasmids and Mutants

The primers used for the construction of plasmids are listed in Table S1. The TALE module targeting the core sequences of the P3 promoter was constructed with the FastTALE^TM^ assembly kit (SIDANSAI, Shanghai, China). The pgpd promoter in the Ptalen R36 (SIDANSAI, Shanghai, China) vector was replaced with the P43 promoter after being digested with restriction enzymes *Asc*I and *Spe*I (Fermentas, ME, USA). The P3 promoter is suitable for *S. cellulosum*. The VP64 element was inserted into the Ptalen R36 vector to replace the *Fok*I element by using the restriction enzyme sites *BamH*I and *Sac*I (Fermentas, ME, USA). The P43 promoter and gRNA sequence targeting sequence in the core sequence of the P3 promoter were fused together by fusing PCR using Prime STAR Max Mix (Takara, Japan); then, the fused fragment was inserted into the pLX-sgRNA vector employing restriction sites *Xho*I and *Nhe*I.

### Introduction of Recombinant TALE-TF Element and dCas9 System

The So ce M4 strain was cultured in a G52 medium for 12 h, and the cells were collected by centrifugation at 8,000 rpm and 4°C. The pellets were washed with ice-cold sterilized water twice to prepare So ce M4 competent cells. The recombinant TALE-TF and dCas9-VP64+PLX-sgRNA vectors were electroporated into the competent cells of So ce M4 under 2.0 KV in a 2.0-cm cuvette. The G52 medium was added for the recovery of So ce M4 competent cells. A G52 plate with 50 μg/ml kanamycin and G52 medium with 100 μg/ml ampicillin were used for the selection of the positive clone containing recombinant TALE-TF element and recombinant dCas9-VP64+PLX-sgRNA vectors, respectively. The genomic DNAs of recombinant So ce M4 clones were extracted using the Genomic DNA extraction kit (Tiangen, Beijing, China) and then used as templates; the primers colE F and colE R were used to amplify colE replicon in Ptalen R36 vector to ensure the introduction of recombinant TALE-TF vector. Primers f1F and f1R, and P43 F and P43 R (Table S1) were used to demonstrate the successful introduction of PLx-sgRNA and pcDNA-dCas9-VP64 vectors.

The total RNAs of the recombinant strain containing TALE-TF element (TALE-So ce M4) and dCas9-VP64 vector (dCas9-So ce M4) were extracted, and the cDNAs were obtained with a 5 × all-in-one reverse transcription mix (Abm, Canada). Then, qRT-PCR was performed by using the primers listed in Table S2 and qRT-PCR mix (Abm, Canada), together with QuantStudio 7 Flex Real-Time PCR System (Thermo Fisher, USA) to detect the relative expression levels of epothilone biosynthetic genes, and GAPDH was used as a reference gene. The qRT-PCR products were identified by using 1% agarose gel.

### Yield Evaluation of Native and Recombinant So ce M4 Strains

Epothilones B and D (Aladin, Shanghai, China) standard at 100, 75, 50, 25, and 10 mg/L were used in the evaluation of epothilones B and D yields in the native and recombinant So ce M4 strains. The native and recombinant So ce M4 strains were inoculated into a G52 medium and cultivated at 28°C. Then, 5% XAD-16 macroporous resin (Amberlite, USA) and 3 mM sodium propionate (Sigma, USA) were added after 3 days of cultivation. After 7 days of cultivation, the resin was eluted using 80% methanol, and the eluent was concentrated to 2 ml with rotary evaporators (EYELA, USA) at 50°C. Then, 10 μl of the concentrated solution and epothilone standards were loaded onto a liquid chromatograph-mass spectrometer (Agilent 6430, USA). The abundances of the ion peaks of epothilones B (508.2, 530.2) and D (492.3, 514.3) were calculated for the evaluation of the yields of epothilones B and D in the native and recombinant So ce M4 strains.

### ChIP Assay of Recombinant TALE-So ce M4 Strain

The ChIP assay of TALE-So ce M4 strain was performed with a ChIP assay kit (Beyotime, China). TALE-So ce M4 strain was cultivated for 24 h at 28°C and 200 rpm and then incubated at 37°C for 10 min. Then, 1% formaldehyde was added for the cross-linking of the target protein and genome DNA. After 3 days of cultivation, glycine solution was added, and PBS containing 1 mM PMSF was added to wash the *S. cellulosum* cells. The So ce M4 cells were collected after centrifugation at 8,000 rpm at 4°C. The SDS lysis buffer was added to the lysis So ce M4 cells, and then the samples were sonicated on ice. The supernatant was collected after centrifugation at 12,000 *g* and then eluted with a ChIP dilution buffer. Thereafter, 70 μl of protein A+G agarose/sperm DNA was added and incubated at 4°C for 30 min. Anti-FLAG mouse monoclonal antibody (Abcam, USA) with dilution of 1:2,000 was added and incubated at 4°C overnight. Then, 60 μl of protein A+G agarose/sperm DNA was added to pull down the primary antibody-TALE protein-DNA complex. The supernatant was removed cautiously after centrifugation, and the precipitate was washed and finally eluted with an elution buffer (1% SDS, 0.1 M NaHCO_3_). The eluate was used as the template, and primer P3 F was used to amplify the desired fragment. The loading buffer was added to the eluate. SDS-PAGE and Western blot analysis using the anti-FLAG antibody were performed to confirm the successful immune precipitation of recombinant TALE protein in the So ce M4 strain.

### Acquisition of Native Regulatory Protein and Recombinant TALE Protein

The core sequence of the P3 promoter was prepared through annealing and then labeled with biotin with a biotin-labeling kit (Beyotime, Beijing, China). The total protein of the native and TALE So ce M4 cells were extracted with a protein extraction kit (Invitrogen, USA). The biotin-labeled P3 promoter with a volume of 10 μl was immobilized on streptavidin beads (Biomag, Wuxi, China), and 10 μl of the extracted proteins was added and incubated at 37°C for 1 h. Then, 50 μl of 95% deionized formamide containing 10 mM EDTA was added to elute the P3 promoter regulatory protein complex.

The recombinant TALE-VP64 vector targeting ATCTTGTGATTCCCCT in the core sequence of the P3 promoter was transformed into BL21(DE3) competent cells and cultivated in an LB medium overnight at 37°C, and the supernatant was collected after sonication. The supernatant was added into FLAG tag magnetic beads and incubated at 37°C for 1 h, and 10 mM glycine solution (pH 2.5) was added to elute the recombinant TALE-VP64 protein. The protein in the elution buffer was identified by SDS-PAGE and Western blot analysis.

### EMSA Assay

The core (5′-TGCGATCTTGTGATTCCCCTTCTGATCTTTAAAATTTCCCGATCCCCCAT-3′) and non-core sequences (TGCGATCTTGTGATTCCCCTTCTGATCTTTAAAATTTCCCGATCC) were labeled with biotin by using the biotin-labeling kit (Beyotime, Beijing, China). The biotin-11-dUTP was added to the 3′ end of the target sequence by using terminal deoxynucleotidyl transferase and incubating at 37°C for 30 min. The sense and antisense strands were incubated at 95°C for 2 min and then cooled to room temperature. The biotin-labeled P3 core and non-core sequences were incubated with recombinant TALE-TF protein and native transcriptional factor P3*Fis* protein at 25°C for 10 min, respectively. The competition EMSA shift experiment was performed by incubating the biotin-labeled probe with P3*Fis* protein and recombinant TALE-VP64-P3 protein together, and the probe without the addition of protein was used as the negative control. A gel-shift buffer was added and identified by using the EMSA gel and then transferred onto nylon membrane under 380 mA for 30 min. Then, the membrane was fixed by using a UV-light cross-linker and blocked with 5% non-fat milk for 30 min at room temperature. The membrane was incubated with biotin antibody (1:5,000) for 40 min at room temperature. The membrane was washed, and the bands were visualized by adding enhanced chemiluminescence substrate according to the instruction of the manufacturers.

## Results

### Identification of Epothilone Biosynthetic Promoter in So ce M4

**Figure 1:**
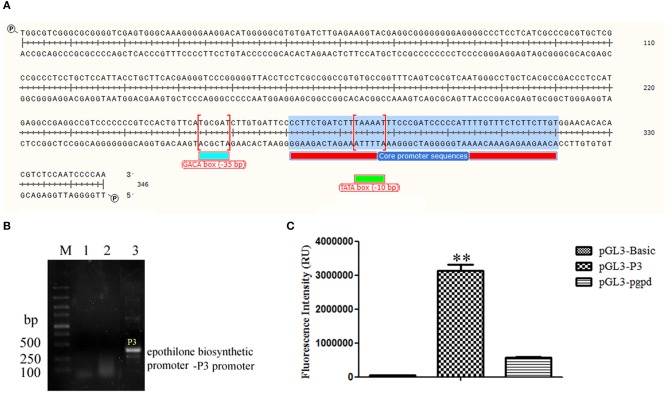
The identification of P3 promoter from *S. cellulosum* So ce M4: **(A)** the sequence of P3 promoter; **(B)** the amplification of P3 promoter using genomic DNAs from different *S. cellulosum* strains: lanes 1–3 were PCR products using genomic DNAs of So ce M1, So ce M6, and So ce M4, respectively; **(C)** the transcriptional activity assay of P3 promoter and pgpd promoter. The transcriptional activity of P3 promoter was significantly higher than that of pgpd promoter. **P < 0.01.

The upper primer 5′-TGGCGTCGGGCGCGGGG-3′ and down primer 5′-TTGGGGATTGGAGACG-3′ were used to amplify the epothilone biosynthetic promoter-P3 promoter with a length of 346 bp. The promoter was located at the upstream of the *epoA* gene, the first gene of the epothilone gene cluster. The core region was predicted at 250–299 bp, the sequence of TAAAAT at the core region of P3 (279–284 bp) was considered TATA box at the −10 bp region, and the sequence of TGCGAT was considered the GACA box of the P3 promoter (Figure 1A). The P3 promoter was then inserted into vector pGL3-basic to initiate the expression of luciferase. The pgpd promoter was also inserted into pGL3-basic as a reference. The supernatant was obtained after the sonication of recombinant BL21 (DE3) strain harboring plasmid pGL3-P3. The fluorescence photometer was used to detect fluorescence intensity. As shown in Figure 1B, the fluorescence intensity of the recombinant strain containing PGL3-P3 was remarkably higher than that in strains harboring the vectors pGL3-basic and pGL3-pgpd. The results provided strong evidence of the function of the P3 promoter in the epothilone biosynthetic cluster of *S. cellulosum* So ce M4 (Figure 1C).

### Construction and Introduction of Recombinant Plasmid

**Figure 2:**
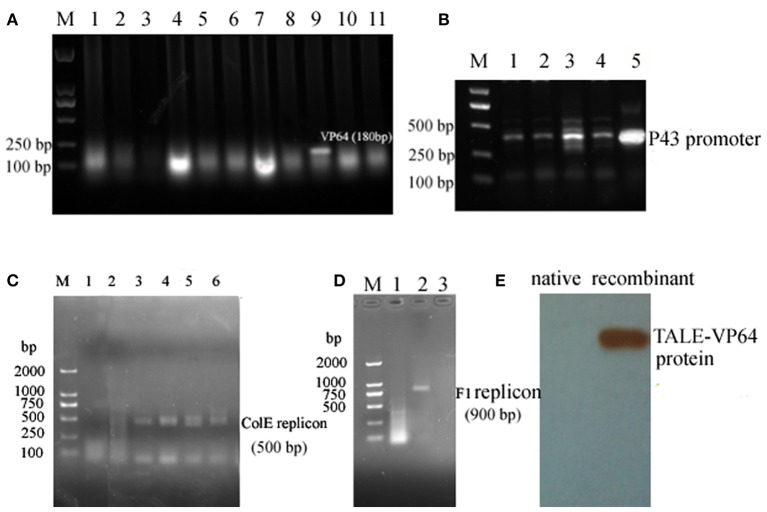
The construction and introduction of recombinant TALE-VP64 and dCas9-VP64 elements: **(A)** the insertion of VP64 element: M. DNA Marker; lanes 1–11 were colony PCR products, lane 9 was the positive clone; **(B)** the insertion of P43 promoter into TALE-VP64 vector and pLX-sgRNA, lanes 1–4 were colony PCR using different colonies as template, lane 5 was positive control; **(C)** the confirmation of the introduction of TALE-VP64-P3 into So ce M4, lane 1 was the native So ce M4, lanes 2–6 were colony PCR products; **(D)** the confirmation of the introduction of dCas9-VP64-P3 into So ce M4: lane 1 was the native So ce M4, lanes 2 and 3 were colony PCR products; **(E)** the confirmation of the introduction of TALE-VP64-P3 vector by Western blot, anti-FLAG antibody was used as a primary antibody.

The core sequence of the P3 promoter was predicted as TGCGATCTTGTGATTCCCCTTCTGATCTTTAAAATTTCCCGATCCCCCAT (http://www.fruitfly.org/seq_tools/promoter.html), and the TALE module targeting sequence ATCTTGTGATTCCCCT was constructed using a fast TALEN assembly kit (SiDansai Biotechnology Ltd. Shanghai). Then, the TALE module was inserted into the vector containing VP64 and FLAG tag (Figure S1). Then, the CMV promoter was replaced by the P43 promoter, which is suitable for *S. cellulosum* So ce M4 (Figure 2A). Then, the P43 promoter was inserted into the pLX-sgRNA and pcDNA-dCas9-VP64 vectors (Figure 2B). The DNA targeting ATCTTGTGATTCCCCT was inserted into the pLX-sgRNA vector. The restriction sites *Xho*I and *Nhe*I were introduced to the P43 promoter and sgRNA scaffold assembled with the target sequence, and then digested and ligated into the pLX-sgRNA vector (Figure S1). The recombinant TALE-VP64-P3 vector and pcDNA-P43-dCas9-VP64+pLX-sgRNA-P3 were electroporated into *S. cellulosum* So ce M4 cells. Then, the positive clone was selected using the G52 plate containing kanamycin and G52 plate containing ampicillin, respectively. The successful amplification of ColE1 (Figure 2C) and f1 replicons (Figure 2D) confirmed the successful introduction of recombinant TALE-VP64 and dCas9-VP64 vector into *S. cellulosum* So ce M4. The total proteins of native and TALE-So ce M4 were extracted, and the anti-FLAG antibody was used to detect the recombinant protein. The band with a molecular weight of 100 kDa confirmed the expression of recombinant TALE protein in TALE-So ce M4, which was detected in native So ce M4 (Figure 2E).

### Expression of Genes Related to Epothilone Biosynthesis in So ce M4

**Figure 3:**
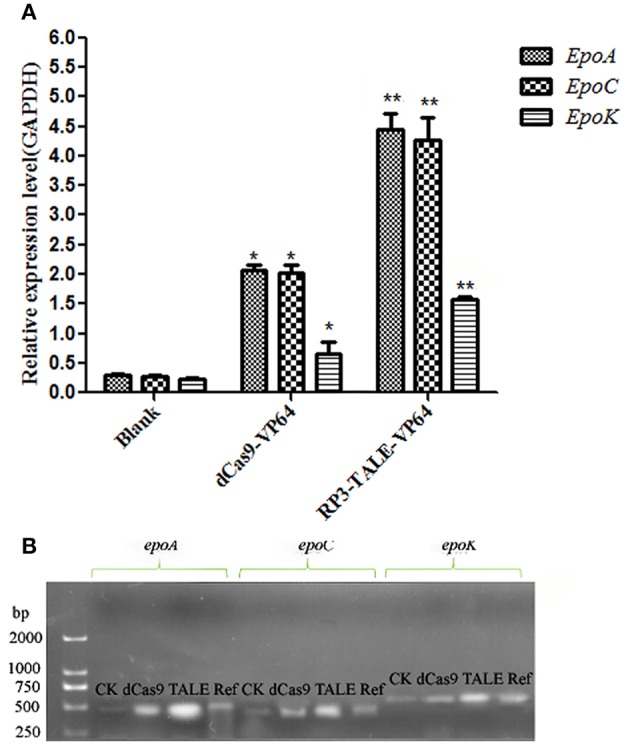
The expression levels of epothilone-biosynthesis-related genes in *S. cellulosum* So ce M4: **(A)** qRT-PCR analysis; **(B)** the agarose gel electrophoresis of qRT-PCR products. CK means qRT-PCR products using cDNA of the native So ce M4 as a template, ref means the qRT-PCR results of GAPDH gene, which was used as a reference gene. dCas9, TALE refer to qRT-PCR products using cDNAs of the dCas9-So ce M4 and TALE-So ce M4 recombinant strains, respectively. *indicates *P* < 0.05, **indicates *P* < 0.01.

The total RNAs of the native and recombinant So ce M4 strains were extracted, and the cDNAs were obtained and used as templates to detect the expression levels of epothilone biosynthesis genes, including *epoA, epoC*, and *epoK*. The results indicated that the expression level of *epoA* in the recombinant So ce strain containing TALE-VP64 elements (TALE-So ce M4) was ~14.89 ± 2.36-fold of that in the native So ce M4 strain. The expression level of *epoA* in the recombinant So ce strain containing dCas9-VP64 (dCas9-So ce M4) was ~6.95 ± 0.38-fold of that in the native So ce M4 strain (Figure 3A). The qRT-PCR products were detected by electrophoresis (Figure 3B). The strongest bands corresponding to *epoA, epoC*, and *epoK* were detected in the TALE-So ce M4 strain, and the weakest was detected in the native So ce M4. The remarkably much higher expression level of epothilone biosynthesis-related genes in the recombinant So ce M4 strains as compared with that in the original So ce M4 strains suggested that the introduction of TALE-TF and dCas9-VP64 can upregulate the biosynthesis of epothilones in So ce M4 strains.

### Improvement in the Epothilone Yields of the Recombinant So ce M4 Strains

**Figure 4:**
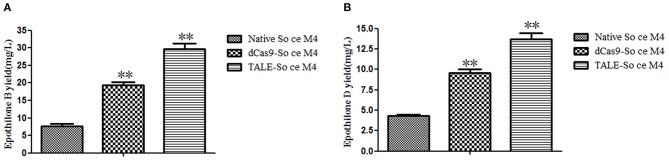
The epothilone yields in different So ce M4 strains: **(A)** epothilone B yields in different So ce M4 strains; **(B)** epothilone D yields in different So ce M4 strains. **indicates *P* < 0.01.

The secondary products of the So ce M4 strains were extracted by using methanol and detected by HPLC-MS. The extract ion chromatographs of epothilones B and D in different So ce M4 strains were acquired. The abundance of epothilone B peaks (508.3, 530.3, and 546.3; Figure S2) and epothilone D peaks (492.2 and 514.2; Figure S3) were calculated for the evaluation of epothilones B and D yields in the So ce M4 strains. When epothilones B and D were used as references, the epothilone B yields in native, dCas9-, and TALE-So ce M4 were 7.64 ± 1.18, 19.33 ± 1.56, and 29.72 ± 2.66 mg/L, respectively. The introduction of dCas9-VP64 and TALE-VP64 elements into the So ce M4 strain improved epothilone B yield by 1.53- and 2.89-fold, respectively. The epothilone D yields in the native, dCas9-, and TALE-So ce M4 were 4.35 ± 0.16, 9.22 ± 1.32, and 13.83 ± 1.67 mg/L, respectively (Figure 4). The TALE-TF element and dCas9-VP64 element improved the epothilone D yield in So ce M4 1.12- and 2.18-fold, respectively. The lower increase in epothilone D yield as compared with that in epothilone B is due to the increased expression level of *epoF* in the dCas9- and TALE-So ce M4 strains, which are responsible for the conversion of epothilone D into epothilone B.

### ChIP Assay of Recombinant TALE-So ce M4 Strain

**Figure 5:**
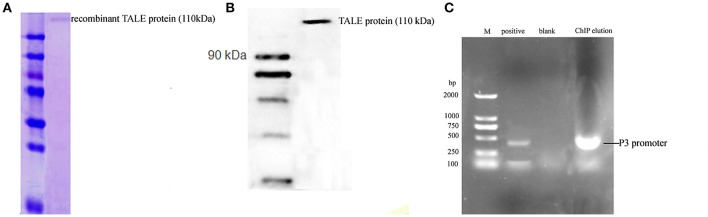
The ChIP assay of *S. cellulosum* So ce M4 containing recombinant TALE-VP64 vector: **(A)** the SDS-PAGE analysis of ChIP eluate; **(B)** the Western blot analysis of ChIP eluate; **(C)** the verification of the presence of P3 promoter in ChIP eluate: M. DNA marker, lanes 1–3 were positive control, blank control, and PCR product using ChIP eluate as a template.

The anti-FLAG primary antibody was used to pull down the recombinant TALE-protein and combined DNA complex. The molecular weight of the obtained protein was ~110 kDa, as estimated by SDS-PAGE (Figure 5A). The result of the Western blot using the anti-FLAG antibody indicated that the obtained band was the TALE-P3 protein, which was not obtained in the native So ce M4 strain. The obtained protein-DNA complex was used as the template (Figure 5B), P3 F was used as the primer, and a fragment with a length of 350 bp approximately corresponding to the P3 promoter was detected (Figure 5C). The sequencing result confirmed that the obtained fragment was the P3 promoter, demonstrating the combination of recombinant TALE-P3 protein and the P3 promoter in the recombinant TALE-So ce M4 strain.

### Acquisition of Native Regulatory Protein and Recombinant TALE Protein

**Figure 6:**
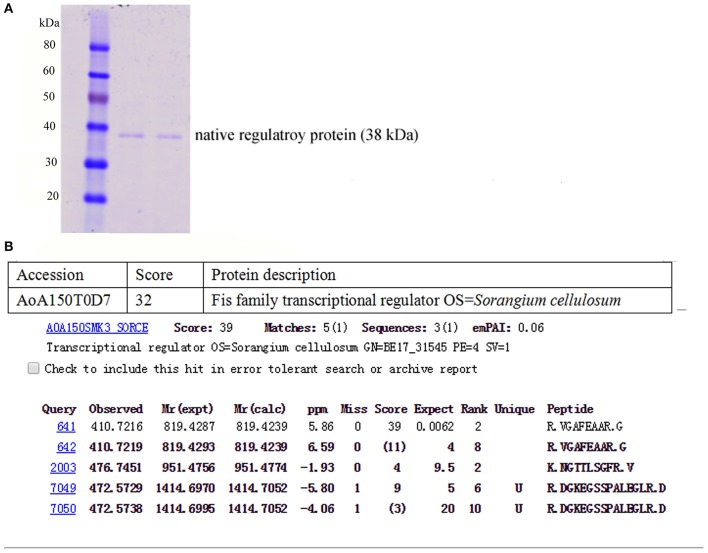
The acquisition of native regulatory protein P3*Fis* in *S. cellulosum* So ce M4: **(A)** the SDS-PAGE analysis of native regulatory protein obtained by P3 probe; **(B)** the amino acid sequencing of native regulatory protein.

The total protein of the So ce M4 stains was extracted at a concentration of 4.15 mg/ml, and the core sequence of the P3 promoter was labeled with biotin. Thus, the native regulatory protein was pulled down by the P3 promoter. The SDS-PAGE results suggested that the obtained regulatory protein was ~38.0 kDa (Figure 6A), and the protein sequencing results showed that the protein sequence matched with an uncharacterized protein in *S. cellulosum* strain So ce 56. The part of digested peptide identified with a shotgun mass spectrometer matched a kind of Fis family transcriptional regulator in the *S. cellulosum* strain So0157-2. This regulator is responsible for the modulation of epothilone biosynthesis in So0157-2. This finding indicated that the obtained protein is a novel kind of regulatory protein for epothilone biosynthesis in So ce M4 strains and named P3*Fis* protein (Figure 6B). The TALE-VP64 element targeting P3 promoter was introduced into the BL21 (DE3) strain, and the recombinant TALE protein was expressed under the P43 constitutive promoter. The P3 promoter was labeled with biotin, and streptomycin affinity beads were used to purify the recombinant TALE-VP64 protein with molecular weight of ~110 kDa, and the acquirement of recombinant TALE-VP64 protein was demonstrated via Western blot analysis using the anti-FLAG primary antibody.

### EMSA Assay of the P3 Promoter and Regulatory Proteins

**Figure 7:**
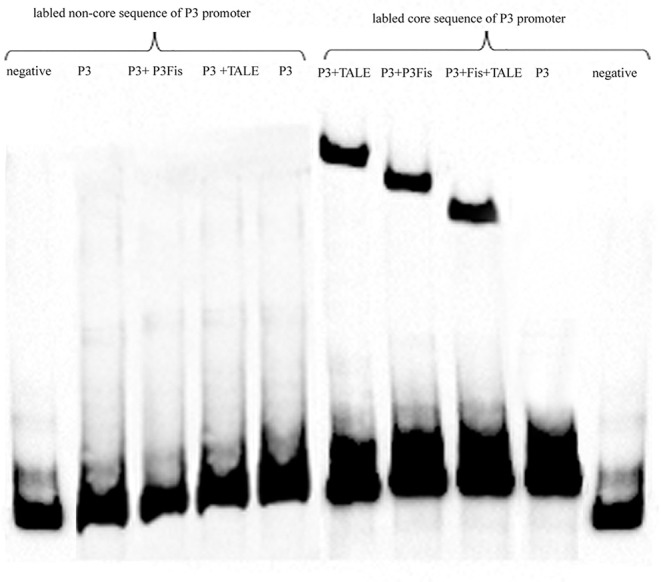
The EMSA assay of native regulatory protein P3*Fis* and recombinant TALE-VP64 protein using biotin labeled core sequences of P3 promoter as probe. The non-core sequences of P3 promoter with the same length was also labeled as a control.

The core sequence of the P3 promoter and other sequences with length of 50 bp (TGGCGTCGGGCGCGGGGTCGAGTGGGCAAAGGGGAAGGACATGGGGGCGT) were labeled with biotin, and the labeled sequences without protein addition was used as a control, as shown in Figure 7. The most remarkable shift was obtained after the addition of the recombinant TALE-TF protein into the P3 core sequence, and a weak shift was observed after the addition of the native regulatory protein. The shift observed after the addition of the mixture of native regulatory protein and recombinant TALE-TF was weaker than that observed after the addition of the regulatory protein alone, suggesting the competitive inhibition of native regulatory protein and recombinant TALE-VP64 protein during the combination of regulatory proteins with the P3 promoter. However, no shift was observed upon the addition of both regulatory proteins into the labeled non-core sequence of the P3 promoter, indicating that the binding sequence of the recombinant TALE-VP64 protein and native regulatory protein was the core sequence of the P3 promoter TGCGATCTTGTGATTCCCCTTCTGATCTTTAAAATTTCCCGATCCCCCAT.

## Discussion

The P3 promoter for epothilone biosynthetic cluster in *S. cellulosum* strain So ce M4 strain was firstly identified. Then, the recombinant TALE-TF element and CRISPR-dCas9-VP64 element targeting the core sequence of the P3 promoter were transformed into the So ce M4 strain for the upregulation of the expression levels of epothilone-biosynthesis-related genes and the improvement of the yields of epothilones B and D. The combination of the recombinant TALE-VP64 element with the P3 promoter *in vivo* and *in vitro* was confirmed by ChIP and EMSA assays, and the binding sequences of the P3 promoter were demonstrated. The native regulatory protein for epothilone biosynthesis in So ce M4 strain was pulled down by using the P3 promoter as the probe, and the competitive inhibition mechanism was demonstrated. The CRISPR/dCas9-VP64 system had been employed in the metabolic engineering of *M. xanthus* (Peng et al., 2018). However, the CRISPR/dCas9 system has not been used in the genetic engineering of *S. cellulosum*, let alone the TALE-TF technology. This study was the first report on the epothilone improvement in *S. cellulosum* strain by using the TALE-TF and CRISPR-dCas9 systems. The regulatory mechanism of TALE-TF elements for epothilone biosynthesis and its competitive effect with native regulatory protein in the *S. cellulosum* strain were also firstly illustrated, thus providing the molecular clues for the further regulation of epothilone biosynthesis in other *S. cellulosum* strains. Hence, this study is of significance and novelty with regard to the improvement of epothilone production in *S. cellulosum* by genetic engineering approaches, especially transcriptional activation.

The epothilones are potent anticancer drugs that bind to tubulin and inhibit the disassembly of microtubules (Bian et al., 2017). The epothilone B derivative ixabepilone has been approved for use in the treatment of breast and ovarian cancer patients, showing remarkable curative effect compared with taxol (Roché et al., 2007; Thomas et al., 2007; Sparano et al., 2010). However, the high cost of ixabepilone due to its complex production procedure limits its clinical application (Sparano et al., 2010). Thus, different approaches have been adopted to improve epothilone production. However, the difficulty in the genetic manipulation of *S. cellulosum*, the unclear genetic information for each *S. cellulosum* strain, and the lack of recognition for epothilone biosynthesis promoter and regulatory proteins impedes the progress of epothilone yield improvement in *S. cellulosum* (Ye et al., 2016). The 440-bp upstream sequence of the epothilone biosynthetic gene cluster from different *S. cellulosum* strains was cloned and identified, and the results indicated that the sequences of different promoters showed high similarity, whereas the transcriptional activities of these promoters remarkably vary probably because of the presence of stem-loop structures in the promoter sequences (Zhu et al., 2013). Different primers were designed to obtain promoters for epothilone biosynthesis in So ce M4, and several sequences were obtained. However, only the essential elements for promoter were found in the P3 promoter, and only the P3 promoter showed strong transcriptional activity. The complete genome of the epothilone-producing strain *S. cellulosum* So0157-2 was sequenced (Schneiker et al., 2007; Han et al., 2013; Bian et al., 2017), the Fis family regulatory protein was identified (Schneiker et al., 2007; Zhu et al., 2012), and the binding activity of this regulatory protein epothilone biosynthesis promoter was confirmed by EMSA assay *in vitro*. The binding activity of Etf1 with epothilone biosynthesis promoter was demonstrated by the co-expression of Etf1 and the promoter ligated with reporter gene EGFP. However, the binding activity of the regulatory protein and promoter has not been confirmed in the So0157-2 strain because of the difficulty in genetically manipulating this strain. Meanwhile, the *in vitro* binding activity of the recombinant regulatory protein TALE-VP64-P3 with epothilone biosynthesis promoter in the *S. cellulosum* So ce M4 strain was firstly confirmed by ChIP assay.

Given that the cell behavior of *S. cellulosum* is complex, leading to increases in the levels of metabolic and regulatory factors and high variability of metabolites and genetic information, genetically manipulating *S. cellulosum* is difficult (Dworkin, 1983; Julien and Shah, 2002). Thus, many researchers have focused on the heterologous expression of the epothilone biosynthetic gene cluster in *M. xanthus* strains. However, the highest yield of epothilone B in recombinant *M. xanthus* strain was <5 mg/L because of the cytotoxicity of epothilones. The disruption of the *epoK* gene in the *M. xanthus* strain can improve the epothilone D yield to 20 mg/L (Julien and Shah, 2002; Bian et al., 2017; Peng et al., 2018). The CRISPR/dCas9 system was introduced into the *M. xanthus* strain, thus improving epothilone B yield 2-fold to 15 mg/L. The epothilone B yield in the *S. cellulosum* strain was higher than 50 mg/L (Gong et al., 2007), indicating that *S. cellulosum* is the optimal candidate host for the production of epothilones. The dcas9 fused with Cuω and the sgRNA targeting sequences with different spacers to the epothilone gene cluster were co-transformed into *M. xanthus*. The results displayed that the sgRNA-targeting spacers of 200 bp at the upstream of the start codon of *epoA* can achieve the highest improvement in epothilone B yield (Peng et al., 2018). Different transcription factors were fused with dCas9 to improve the expression levels of *epoA, B, C, D, E, F*, and *P* genes in *M. xanthus*, and the expression levels of *epoA* and *epoP* were improved ~8- and 12-fold, respectively. The epothilones A and B yields in *M. xanthus* were improved by <2-fold by the CRISPR/dCas9 system, which was less than that the TALE-TF system used in *S. cellulosum* in our study. The results were in accordance with our investigation on *epo* gene expression levels and epothilones yields. Some epothilone biosynthetic genes, which were located far from the P3 promoter, also showed high expression levels; for instance, the expression level of the *epoK* gene in the TALE-So ce M4 was ~7.09 ± 1.25-fold of that in the native So ce M4 strain, which may be due to the presence of other promoters in epothilone biosynthesis gene cluster of *S. cellulosum* So ce M4 besides P3 promoter. The production of epothilones was positive related to the expression levels of various epothilone biosynthetic genes. The low expression levels of some epothilone biosynthetic genes as compared with the expression level of *epoA* and the limited supply of precursors including propanyl COA in the fermentation liquid resulted in low increment in epothilone yield relative to that of the epothilone biosynthetic genes.

The 5′ end of the P3 promoter in *S. cellulosum* was at ~-440 bp, and the core sequence was ~-250 bp. Thus, the core sequence of the P3 promoter was ~200 bp upstream of the epothilone biosynthetic gene cluster in So ce M4. Thus, the approaches using TALE-VP64 and dCas9-VP64 activators targeting the core sequence of the P3 promoter were effective in improving epothilone B yield in the So ce M4 strain. The introduction of recombinant TALE-VP64 protein and dCas9-VP64 improved epothilone B yield 2.89- and 1.53-fold, respectively. The higher increased increment of epothilone B yield by the transformation of the recombinant TALE-TF element into the So ce M4 strain compared with that of the CRISPR-dCas9 elements was probably due to the increased binding activity and specificity of TALE elements with the core sequence of the P3 promoter relative to those of the sgRNA sequence. It was also reported that protein-DNA binding showed higher affinity and specificity than DNA-DNA binding (Ashworth and Baker, 2009).

## Conclusions

In this study, a novel promoter P3 for the epothilone biosynthesis in *S. cellulosum* So ce M4 was acquired, and the transcriptional activity was also verified. The TALE-TF technology and CRISPR/dCas9 technology were firstly used to enhance the biosynthetic efficiency of the epothilone biosynthetic gene cluster, and the introduction of dCas9-VP64 and TALE-VP64 elements into the So ce M4 strain improved epothilone B and epothilone D yields by 1.53- and 2.89-fold and 1.12- and 2.18-fold, respectively. The transcriptional regulatory mechanism of TALE-TF elements and the competitive inhibitory effect with novel native regulatory protein P3*Fis* were revealed by EMSA and ChIP analysis. This is the first report on the transcriptional regulation of the epothilone biosynthetic gene cluster in *S. cellulosum* using the TALE-TF and dCas9-VP64 systems, as well as the regulatory mechanism of the TALE-TF system in *S. cellulosum*. Our investigation would open a new avenue for the yield improvement of anticancer drug epothilones in the native host *S. cellulosum* by transcription activation approaches, thus promoting the applications of epothilones and their derivatives in the biomedical industry.

## Data Availability Statement

The datasets generated for this study are available on request to the corresponding author and Supplementary Materials.

## Author Contributions

WY and WZ designed the experiments. WY, TL, MZ, ZH, YK, YC, and SL conducted experiments. SL and HL provided materials. WY, TL, and WZ analyzed data. WY wrote the manuscript, which was revised by WZ. WZ directed the research.

### Conflict of Interest

The authors declare that the research was conducted in the absence of any commercial or financial relationships that could be construed as a potential conflict of interest.

## Supplementary Material

Figure S1 The activation of epothilone biosynthetic cluster using TALE-VP64 and dCas9-VP64 system in *S. cellulosum* So ce M4.

Figure S2 The detection of epothilone B yields in different So ce M4 strains by LC-MS: **(A)** epothilone standard; **(B)** native So ce M4; **(C)** RP3-So ce M4; **(D)** dCas9-So ce M4. The MS peak of 508.3 was corresponding to epothilone B, and the peak of 508.3 was extracted from the chromatography of different So ce M4 strains.

Figure S3 The detection of epothilone D yields in different So ce M4 strains by LC-MS: **(A)** epothilone standard; **(B)** native So ce M4; **(C)** RP3- So ce M4; **(D)** dCas9-So ce M4. The MS peak of 492.2 was corresponding to epothilone B, and the peak of 492.2 was extracted from the chromatography of different So ce M4 strains.

Table S1 Primers used for the construction of recombinant TALE-TF vector and dCas9-VP64 vectors.

Table S2 Primers used for the qRT-PCR of genes related to the epothilone biosynthesis in *S. cellulosum* So ce M4.

## Abbreviations

CHIP: Chromatin immunoprecipitation
CRISPR: Clustered regularly interspaced short palindromic repeats
ECL: Enhanced chemiluminescence
EMSA: Electrophoretic mobility shift assay
LC-MS: Liquid chromatograph-mass spectrometer
TALE-TF: Transcription activator-like effectors-Trancriptional factor.
