# Supplementary figures and images for: An Easy and Efficient Strategy for the Enhancement of Epothilone Production Mediated by TALE-TF and CRISPR/dcas9 Systems in Sorangium cellulosum
Source: Front Bioeng Biotechnol. 2019 Nov 26;7:334. doi: 10.3389/fbioe.2019.00334 (PMC6988809; doi:10.3389/fbioe.2019.00334)

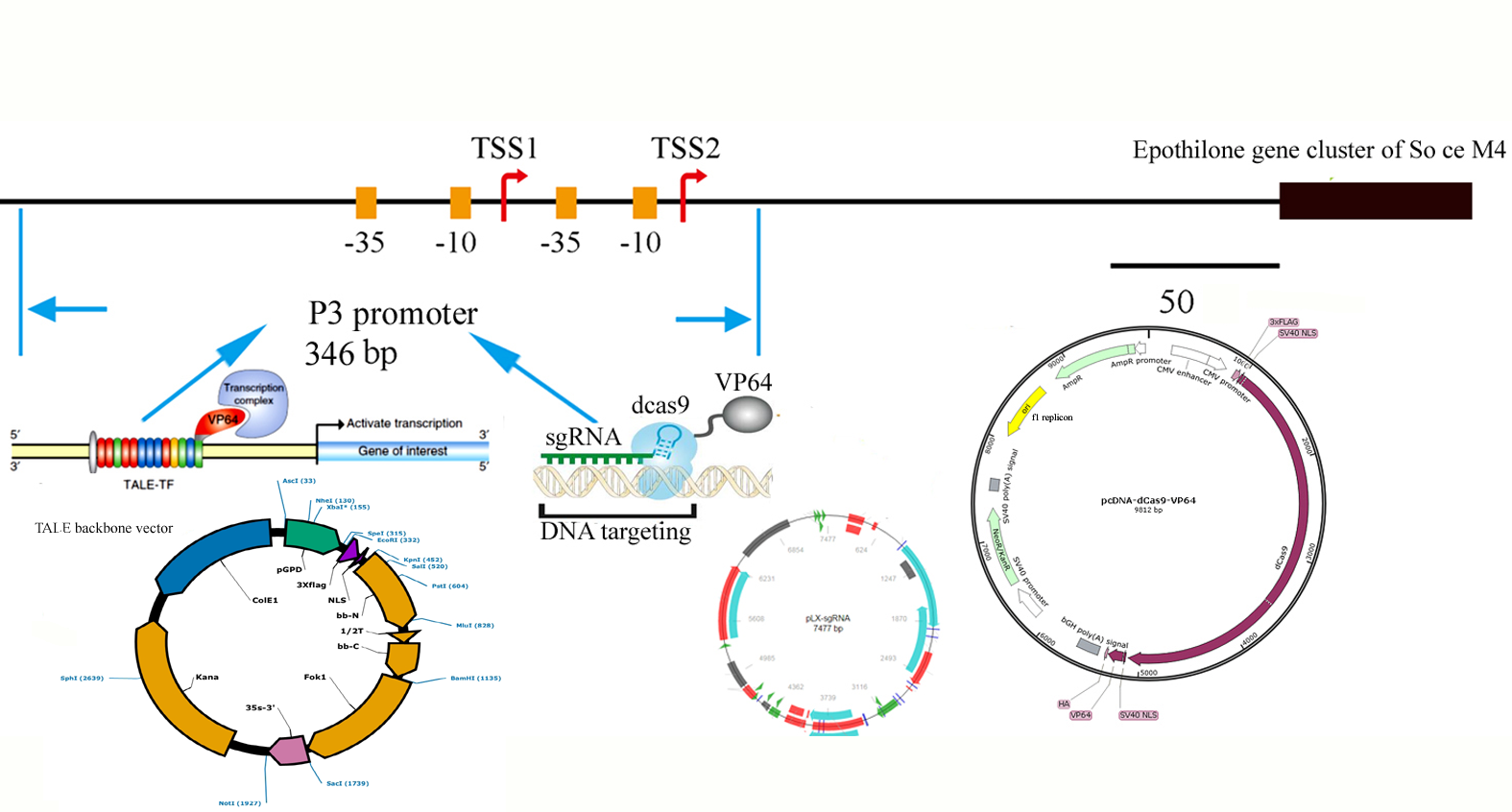

Supplement: Figure S1 — The activation of epothilone biosynthetic cluster using TALE-VP64 and dCas9-VP64 system in S. cellulosum So ce M4. [file Image_1.TIF]

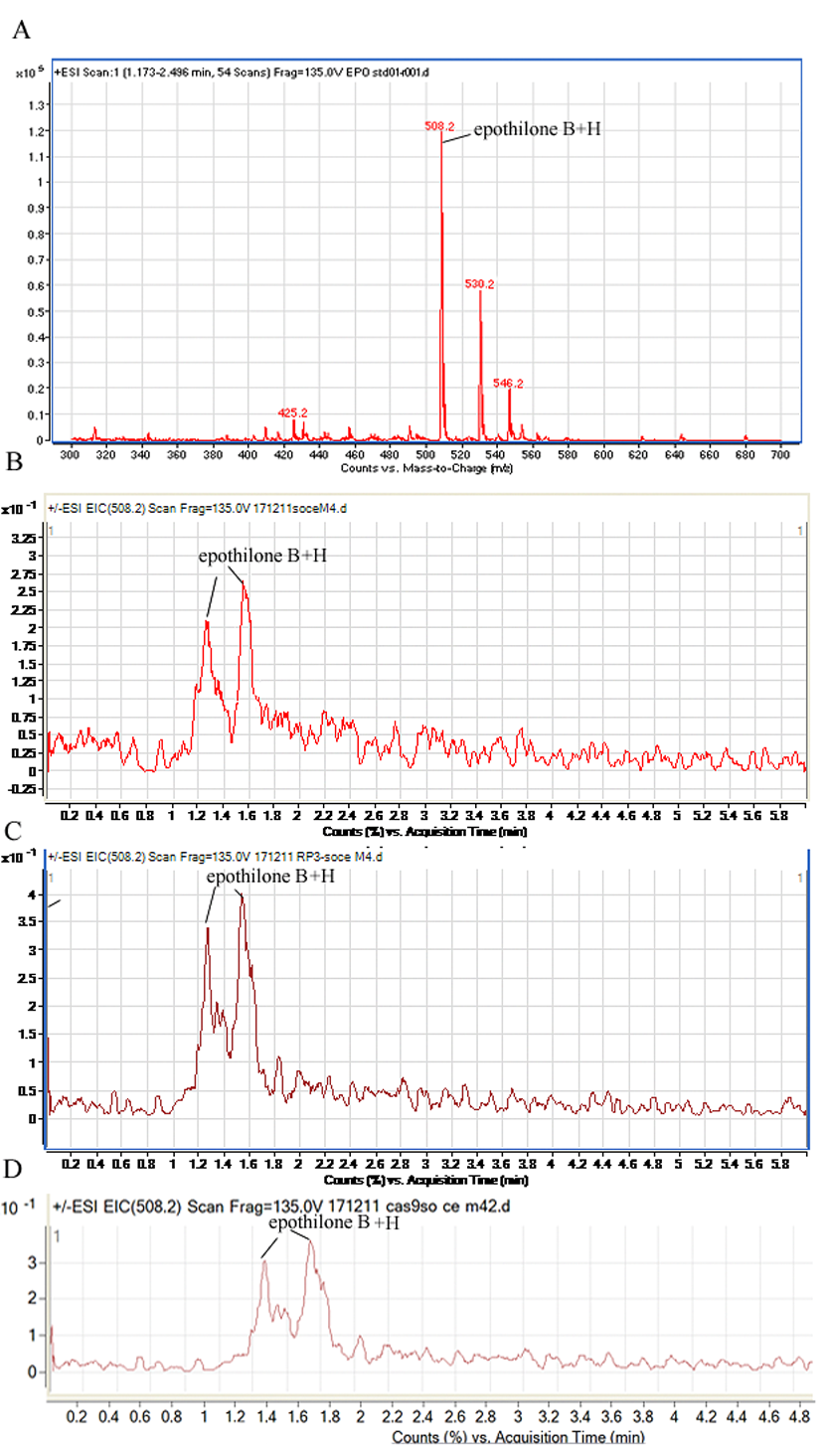

Supplement: Figure S2 — The detection of epothilone B yields in different So ce M4 strains by LC-MS: (A) epothilone standard; (B) native So ce M4; (C) RP3-So ce M4; (D) dCas9-So ce M4. The MS peak of 508.3 was corresponding to epothilone B, and the peak of 508.3 was extracted from the chromatography of different So ce M4 strains. [file Image_2.TIF]

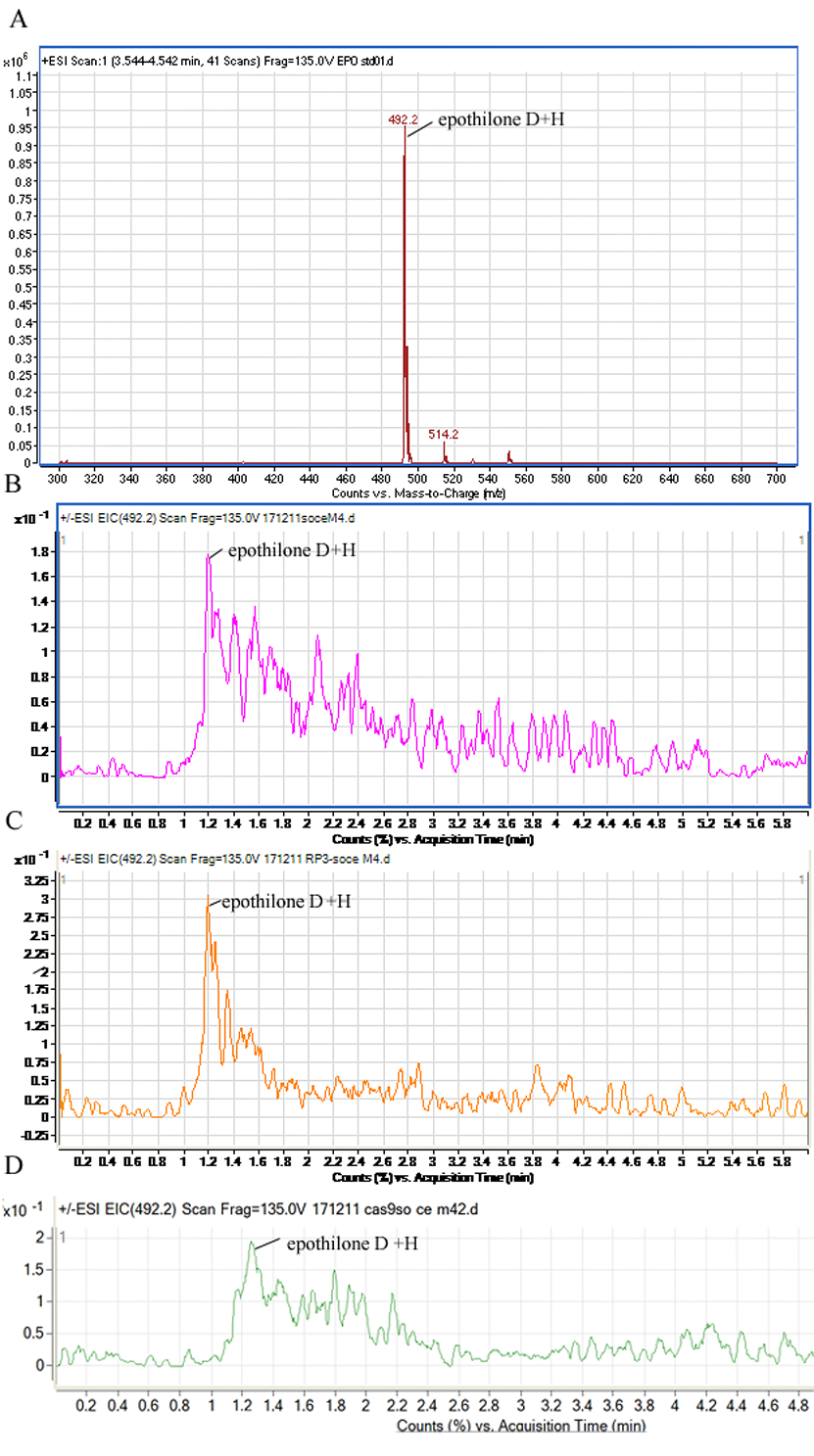

Supplement: Figure S3 — The detection of epothilone D yields in different So ce M4 strains by LC-MS: (A) epothilone standard; (B) native So ce M4; (C) RP3- So ce M4; (D) dCas9-So ce M4. The MS peak of 492.2 was corresponding to epothilone B, and the peak of 492.2 was extracted from the chromatography of different So ce M4 strains. [file Image_3.TIF]
