# Supplementary material for: An Easy and Efficient Strategy for the Enhancement of Epothilone Production Mediated by TALE-TF and CRISPR/dcas9 Systems in Sorangium cellulosum
Source: Front Bioeng Biotechnol. 2019 Nov 26;7:334. doi: 10.3389/fbioe.2019.00334 (PMC6988809; doi:10.3389/fbioe.2019.00334)
Supplement: Table S1 — Primers used for the construction of recombinant TALE-TF vector and dCas9-VP64 vectors. [file Table_1.docx]

Table S1 Primers used for the construction of recombinant TALE-TF vetor and dCas9-VP64 vectors

| Primers | Sequences(5’-3’) |
| --- | --- |
| VP64 F | CGCGGATCCGAACTTCCCATCGAGTCGC |
| VP64 R | CGAGCTCTTAGGGGGGGGGGAGGGAG |
| P43 TALE F | TTGGCGCGCCGGCGGAATTCTGATAGGTG |
| P43 TALE R | CGGACTAGTCGCAAGCTTAACCTTGAAG |
| P43 CRISPR F | AAACTCGAGGGCGGAATTCTGATAGGTG |
| sgRNA R | AAAGCTAGCTAATGCCAACTTTGTACAAGAAAGCTG |
| P3 F | CCTTCTGATCTTTAAAATTTCCCGATCCCCCATTTTGTTTCTCTTCTTGT |
| P3 R | ACAAGAAGAGAAACAAAATGGGGGATCGGGAAATTTTAAAGATCAGAAGG |
| ColE F | TGAGATCCTTTTTTTCTGCGCGTAA |
| ColE R | TTTCCATAGGCTCCGCCCCCCTGACG |
| f1 F | CTTCTGAGGCGGAAAGAACCAG |
| f1 R | CCATCTTGTTCAATCATGCGAAAC |

Note: P43 CRISPR F and P3 R were used to amplify fragment 1 using P43 promoter as a template, P3 F and sgRNA were used to amplify fragment 2 using PLX-sgRNA as a template. Fragment 1 and 2 were used as a mix template, P43 F and sgRNA R were used as primers to obtain fragment 3 fusing P43 and gRNA targeting P3 promoter together, the fragment was ligated into PLX-SgRNA using restriction enzymes *Xho*I and *Nhe*I. The T7 promoter in the pCDNA-dCas9-VP64 is also suitable for the expression of dCas9 in *S. cellulosum* So ce M4.
