# Supplementary material for: An Easy and Efficient Strategy for the Enhancement of Epothilone Production Mediated by TALE-TF and CRISPR/dcas9 Systems in Sorangium cellulosum
Source: Front Bioeng Biotechnol. 2019 Nov 26;7:334. doi: 10.3389/fbioe.2019.00334 (PMC6988809; doi:10.3389/fbioe.2019.00334)
Supplement: Table S2 — Primers used for the qRT-PCR of genes related to the epothilone biosynthesis in S. cellulosum So ce M4. [file Table_2.DOCX]

Table S2 Primers used for the qRT-PCR of genes related to the epothilone biosynthesis in *S. cellulosum* So ce M4.

| Genes | Sequences(5’-3’) |
| --- | --- |
| *epoA* F | CTGGCTGGTGGGGTATCGCT |
| *epoA* R | TGCTGAAGGGACAAGACGAC |
| *epoC* F | GAACCTCCACGAGCACCCAG |
| *epoC* R | TGGCAGACCCAAGGATGACC |
| *epoK* F | ACTCGCATCTCAATCCGCTG |
| *epoK* R | CGGCACTTCTTCCGACGTTA |
